# The Complete Mitochondrial Genome of *Meloidogyne graminicola* (Tylenchina): A Unique Gene Arrangement and Its Phylogenetic Implications

**DOI:** 10.1371/journal.pone.0098558

**Published:** 2014-06-03

**Authors:** Longhua Sun, Kan Zhuo, Borong Lin, Honghong Wang, Jinling Liao

**Affiliations:** 1 Laboratory of Plant Nematology, South China Agricultural University, Guangzhou, China; 2 Guangdong Province Key Laboratory of Microbial Signals and Disease Control, South China Agricultural University, Guangzhou, China; INRA, France

## Abstract

*Meloidogyne graminicola* is one of the most economically important plant parasitic-nematodes (PPNs). In the present study, we determined the complete mitochondrial (mt) DNA genome sequence of this plant pathogen. Compared with other PPNs genera, this genome (19,589 bp) is only slightly smaller than that of *Pratylenchus vulnus* (21,656 bp). The nucleotide composition of the whole mtDNA sequence of *M. graminicola* is significantly biased toward A and T, with T being the most favored nucleotide and C being the least favored. The A+T content of the entire genome is 83.51%. The mt genome of *M. graminicola* contains 36 genes (lacking *atp8*) that are transcribed in the same direction. The gene arrangement of the mt genome of *M. graminicola* is unique. A total of 21 out of 22 tRNAs possess a DHU loop only, while *tRNA^Ser(AGN)^* lacks a DHU loop. The two large noncoding regions (2,031 bp and 5,063 bp) are disrupted by *tRNA^Ser(UCN)^*. Phylogenetic analysis based on concatenated amino acid sequences of 12 protein-coding genes support the monophylies of the three orders Rhabditida, Mermithida and Trichinellida, the suborder Rhabditina and the three infraorders Spiruromorpha, Oxyuridomorpha and Ascaridomorpha, but do not support the monophylies of the two suborders Spirurina and Tylenchina, and the three infraorders Rhabditomorpha, Panagrolaimomorpha and Tylenchomorpha. The four Tylenchomorpha species including *M. graminicola*, *P. vulnus*, *H. glycines* and *R. similis* from the superfamily Tylenchoidea are placed within a well-supported monophyletic clade, but far from the other two Tylenchomorpha species *B. xylophilus* and *B. mucronatus* of Aphelenchoidea. In the clade of Tylenchoidea, *M. graminicola* is sister to *P*. *vulnus*, and *H. glycines* is sister to *R. similis*, which suggests root-knot nematodes has a closer relationship to Pratylenchidae nematodes than to cyst nematodes.

## Introduction

Nematodes are a diverse group of bilateral animals, including free-living, insect-parasitic, animal-parasitic and plant-parasitic forms. Over 4,100 species of plant-parasitic nematodes (PPNs) have been reported to date [1], which cause tremendous economic losses to agriculture worldwide. It is estimated that total losses caused by PPNs are $80 billion annually [2]. PPNs usually include migratory ectoparasites, migratory endoparasites, semi-endoparasites and sedentary endoparasites. Due to their economic importance and diversified life styles, PPNs have always received sufficient attention regarding their taxonomy and evolution [3]. The most economically damaging PPNs, such as root-knot nematodes (RKN, *Meloidogyne*), cyst nematodes (CN, *Globodera* and *Heterodera*), root lesion nematodes (*Pratylenchus*) and burrowing nematodes (*Radopholus similis*), are traditionally considered to belong to the order Tylenchida [4], [5]. More recently, molecular data in combination with morphological features have been used to help resolve nematode classification problems. De Ley and Blaxter [6] provided one classification of nematodes that is used in the present study, in which the Tylenchida PPNs mentioned above were placed in the infraorder Tylenchomorpha. Moreover, Tylenchomorpha belongs to the suborder Tylenchina within the order Rhabditida. However, the molecular information supporting classification of PPNs is currently limited; mining of molecular data from more different species is urgently needed for improved classification.

The most economically important PPNs, RKN and CN, are both sedentary endoparasitic nematodes that induce complex feeding structures in the roots of their hosts [7], the classification of RKN and CN have always been gained prominence. Two main classifications have been suggested for RKN and CN. Specifically, Maggenti [4] placed RKN in the subfamily Meloidogyninae and CN in the subfamily Heteroderinae, and both subfamilies were placed within the family Heteroderidae belonging to the suborder Tylenchina, whereas Siddiqi [5] placed RKN and CN in separate families (resp. Meloidogynidae and Heteroderidae) within the suborder Hoplolaimina. However, several molecular phylogenies based on rDNA show that RKN are closely related to some Pratylenchidae nematodes, while CN are closely related to Hoplolaimidae [3], [8]–[11].

The mitochondrial (mt) genomes of animals are approximately 14 to 20 kb in size and usually comprise 36–37 genes, including 12–13 protein-coding genes (*atp6*, *atp8*, *cox1-3*, *cob*, *nad1-6* and *nad4L*), two ribosomal RNA genes (small and large subunit ribosomal RNA [*rrnS* and *rrnL*]) and 22 transfer RNA (tRNA) genes. No introns are found within these genes, and only limited spacer regions exist between genes [12]–[14]. Thus, mtDNA has been widely used not only for molecular phylogenetic relationship and evolutionary studies, but also for species identification and genetic investigations due to the abundance, small size, maternal inheritance, relatively rapid evolutionary rate and lack of genetic recombination of mtDNA [15]–[18].

In *Meloidogyne*, the mitochondrial DNA segment including 3′ end of *cox2*, the complete *tRNA^His^* and 5′ portion of *rrnL* genes plays an increasingly important role in the differentiation of major *Meloidogyne* species [19], [20]. In addition, some mitochondrial DNA segments such as 63-bp variable number tandem repeat (VNTR) and the region from the partial *cox2* to the partial *cob* genes have often been used to study population biology [21]–[23]. In recent years, increasing numbers of complete mt genomes of nematodes have been sequenced. To date, more than 70 complete mtDNA sequences of nematode species have been deposited into GenBank (http://www.ncbi.nlm.nih.gov, last accessed January 20, 2014). Most mt genomes are derived from the class Chromodorea species, only thirteen mt genomes from the class Enoplean nematodes. Among these, only five entire mtDNA genome sequences derived from PPNs, including *Radopholus similis*, *Bursaphelenchus xylophilus*, *Pratylenchus vulnus* and *B. mucronatus* in the Chromadorea and *Xiphinema americanum* belonging to the Enoplean, have been elucidated [17], [24]–[26]. In addition, partial mt genomic sequences from PPNs *Globodera rostochiensis*, *G. pallid* and *Heterodera glycines* are also present in GenBank [27]–[29]. These mtDNA genomes provide new insights into the phylogeny of nematodes. Recently, Sultana *et al*. [17] conducted phylogenetic analysis as inferred from 41 nematode mt genomes, including four from Tylenchomorpha PPNs. Liu *et al*. [18] analyzed the phylogenetic relationships of 65 nematode species including two PPNs of Tylenchomorpha using mt genome data. However, the most important Tylenchomorpha nematode, RKN, was not included in these two phylogenetic analyses because no entire mt genomes of *Meloidogyne* are currently available in GenBank, although a partial nucleotide sequence of mtDNA from *M. javanica* has been reported [30]. Hence, it is necessary to obtain entire mt genome sequences of RKN to assess the phylogenetic position of *Meloidogyne* in relation to other Tylenchomorpha nematodes and conduct evolutionary studies.

In this study, we determined the complete mtDNA sequence of *M. graminicola*. *M. graminicola* is one of the most important *Meloidogyne* species and is a major species that attacks rice; *M. graminicola* can cause yield losses of up to 87% [7]. In addition, we used these data to infer the phylogenetic relationships among the major groups of Chromadoreans including *Meloidogyne*.

## Results and Discussion

### General features of the mt genome of *M. graminicola*

**Figure 1:**
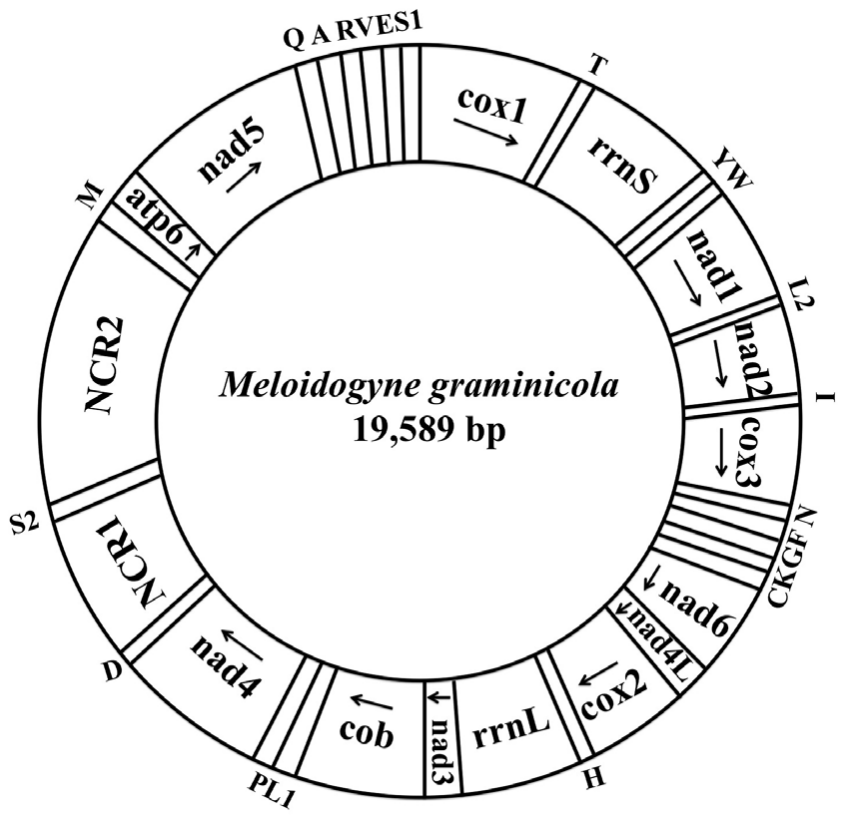
Arrangement of the mitochondrial genome of *Meloidogyne graminicola*. Gene scaling is only approximate. All genes are coding by the same DNA strand, and the arrow indicates the direction of transcription. All protein-coding genes have standard nomenclature. All tRNA genes follow the one-letter amino acid code; L1/L2 and S1/S2 indicate tRNA genes for *tRNA^Leu(CUN)^*/*tRNA^Leu(UUR)^* and *tRNA^Ser(AGN)^*/*tRNA^Ser(UCN)^*, respectively. “NCR1” refers to a small noncoding region and “NCR2” refers to a large noncoding region.

The complete mtDNA sequence of *M. graminicola* is 19,589 bp in length (Figure 1) and has been deposited into GenBank (Accession number KJ139963). The mt genome of *M. graminicola* is larger than those of four PPNs reported to date, i.e., *X. americanum* (12,626 bp) [24], *B. mucronatus* (14,583 bp) [26], *B. xylophilus* (14,778 bp) [17] and *R. similis* (16,791 bp) [25] but slightly smaller than the mt genome of *P. vulnus* (21,656 bp) [17]. The larger sizes of the mt genomes of *M. graminicola* and *P. vulnus* are partly due to the presence of long noncoding regions.

The mtDNA of *M. graminicola* contains 12 protein-coding genes (PCGs), i.e., *cox1-3*, *nad1-6*, *nad4L*, *atp6* and *cob*, 22 tRNAs and two ribosomal RNA genes (*rrnL* and *rrnS*) but lacks an *atp8* gene (Figure 1; Table 1). All genes are transcribed in the same direction, which is consistent with previously reported Chromadorean four PPNs, but distinct from Enoplean *X. americanum*. In *X. americanum*, nine PCGs, ten tRNA and two rRNA genes are located on the GT-rich strand, whereas three other PCGs and seven tRNA genes are located on the AC-rich strand [24]. The arrangement of all 36 genes of *M. graminicola* mtDNA is different from those of nematodes with complete sequences in NCBI. We compared the arrangement of genes in the mtDNA of *M. graminicola* with that shown in the mtDNA draft map of *M. javanica*, comprising 12 PCGs and three tRNAs [30], and found that the order of 12 PCG genes is the same but the location of tRNA genes is different. However, since the complete mtDNA sequence of *M. javanica* is not available in GenBank and the complete tRNA information is not available, we could not perform a detailed comparison of the arrangement of genes between the two RKN species.

**Table 1:** Organization of the *Meloidogyne graminicola* mitochondrial genome.

Gene	Location (bp)	Size (bp)	Start Codon	Stop Codon	Anticodon	Intergenic Nucleotides^a^
*cox1*	1–1554	1554	ATT	TAA		+5
*tRNA^Thr^*	1524–1582	59			TGT	−31
*rrnS*	1583–2178	596				0
*tRNA^Tyr^*	2179–2231	53			GTA	0
*tRNA^Trp^*	2232–2284	53			TCA	0
*nad1*	2282–3166	885	TTG	TAA		−3
*tRNA^Leu(UUR)^*	3134–3186	53			TAA	−33
*nad2*	3221–4027	807	ATT	TAA		+34
*RNA^Ile^*	4033–4085	53			GAT	+5
*cox3*	4084–4854	771	TTG	TAA		−2
*tRNA^Asn^*	4851–4904	54			GTT	−4
*tRNA^Phe^*	4905–4960	56			GAA	0
*tRNA^Gly^*	4961–5013	53			TCC	0
*tRNA^Lys^*	5012–5066	55			TTT	−2
*tRNA^Cys^*	5064–5119	56			GCA	−3
*nad6*	5166–5555	390	ATT	TAG		+46
*nad4L*	5546–5791	246	ATT	TAA		−10
*cox2*	5775–6449	675	ATT	TAA		−17
*tRNA^His^*	6440–6493	54			GTG	−10
*rrnL*	6494–7308	815				0
*nad3*	7309–7614	306	ATT	TAG		0
*cob*	7633–8667	1035	ATA	TAG		+21
*tRNA^Leu(CUN)^*	8671–8727	57			TAG	+3
*tRNA^Pro^*	8725–8777	53			TGG	−3
*nad4*	8775–9944	1170	ATA	TAG		−3
*tRNA^Asp^*	9949–10002	54			GTC	+4
*NCR1*	10003–12033	2031				0
*tRNA^Ser(UCN)^*	12034–12089	56			TGA	0
*NCR2*	12090–17152	5063				0
*tRNA^Met^*	17153–17208	56			CAT	0
*atp6*	17265–17801	537	TTT	TAA		+56
*nad5*	17780–19282	1503	ATA	TAA		−22
*tRNA^Gln^*	19281–19334	54			TTG	−2
*tRNA^Ala^*	19335–19387	53			TGC	0
*tRNA^Arg^*	19388–19440	53			TCG	0
*tRNA^Val^*	19429–19481	53			TAC	−12
*tRNA^Glu^*	19480–19532	53			TTC	−2
*tRNA^Ser(AGN)^*	19531–19584	54			TCT	−2

a: Indicates gap nucleotides (positive value) or overlapping nucleotides (negative value) between two adjacent genes;

NCR: Noncoding region.

**Figure 2:**
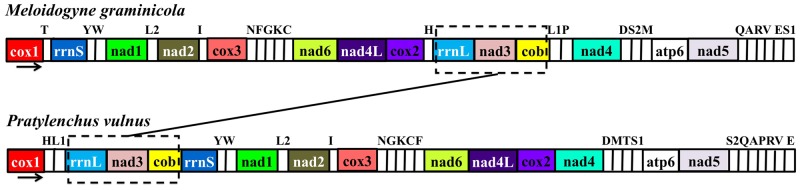
Comparison of mitochondrial gene arrangements between *Meloidogyne graminicola* and *Pratylenchus vulnus*. Gene and genome size are not to scale. The noncoding region (NCR) is not indicated. Arrows below the gene order map indicate the direction of transcription of genes. Genes involved in the rearrangements are shown in dashed boxes.

In contrast to Enoplean mitochondria, which show much greater variation in gene arrangement encoded by both strands, Chromadorean mitochondria share relatively conserved synteny and genes that are encoded on the same strand (Figure S1 in File S1). The most common type of gene arrangement is GA9 which has been found in 18 nematode species among 50 nematode species cited here. Compared to the GA9 type, the gene arrangement of *M. graminicola* shared three small blocks (*nad6-nad4L*, *cox2*-*trnH*-*rrnL*-*nad3* and *cob-trnL1*). If tRNA genes are ignored, the gene arrangements of *M. graminicola* and *P. vulnus* are most similar; two large blocks (*rrnS-nad1-nad2-cox3-nad6-nad4L-cox2* and *nad4-atp6-nad5-cox1*) are identical between them. Only a small block, “*rrnL-nad3-cob*”, was translocated into the middle of *cox2* and *nad4*, which resulted in a novel gene order in *M. graminicola* (Figure 2).

Seventeen overlaps exist in the mtDNA of *M. graminicola*, with overlapping regions ranging from 2 to 33 bp in length; the largest overlap was detected between *nad1* and *tRNA^Leu(UUR)^*. Such overlaps of a few genes are a general feature of metazoan mtDNA [13]. Like *P. vulnus*, the mtDNA of *M. graminicola* contains a long AT-rich noncoding region (NCR). However, in *M. graminicola*, the AT-rich region is located between *tRNA^Asp^* and *tRNA^Met^* and is separated by *tRNA^Ser(UCN)^* and includes a short noncoding region (NCR1, 2,031 bp) and a long noncoding region (NCR2, 5,063 bp).

The nucleotide compositions of the entire mtDNA sequence of *M. graminicola* is significantly biased toward A and T, with T being the most favored nucleotide and C being the least favored, in accordance with the mt genomes of *B. mucronatus*, *B. xylophilus*, *P. vulnus* and *R. similis*, except for *X. americanum* (Table S1 in File S1). The A+T content is 83.51% for *M. graminicola*, with contents of 31.33%, 52.18%, 5.62% and 10.87% for A, T, C and G, respectively. The lowest A+T content of PCGs is 75.36% (in *cox1*), while the highest is 90.51% (in *nad6*). The A+T content of *rrnS*, *rrnL* and the NCR is 84.23%, 87.24% and 82.59%, respectively.

The bias of the base composition of each strand can usually be described in terms of skewness [31], which is calculated as (A% - T%)/(A%+T%) and (G% - C%)/(C%+G%), respectively. The AT- and GC-skewness of the whole mtDNA sequences of PPNs were calculated (Table S1 in File S1). The entire mtDNA sequence of *M. graminicola* is significantly biased toward T (AT skew  = −0.250 and GC skew  = 0.318). The pattern of skew values of *M. graminicola* is highly congruent with those observed in the mtDNA sequences of the five PPNs examined except for *X. americanum*. Hassanin *et al*. [32] suggested that GC skew values are the best indicator of strand asymmetry. In the entire mtDNA sequences of PPNs reported to date, only the GC skew of *X. americanum* has a negative value because the C content of the mtDNA of *X. americanum* is higher than its G content.

### Protein-encoding genes and codon usage

We determined the boundaries between PCGs of the mtDNA of *M. graminicola* by aligning its sequence with that of other nematodes reported to date and identifying the translation initiation and termination codons. In *M. graminicola*, of the 12 PCGs, six (*cox1*, *cox2*, *nad2*, *nad3*, *nad4L* and *nad6*) appear to use ATT as the putative start codon, while three (*cob*, *nad4* and *nad5*) start with ATA, two (*nad1* and *cox3*) start with TTG, and *atp6* starts with TTT. These four start codons have been reported as typical start codons in the mtDNA of other nematodes (Table S2 in File S1). The most frequently used start codon in *M. graminicola*, ATT, is also the most commonly used start codon in *B. xylophilus* (nine PCGs), *H. glycines* (eight PCGs) and *B. mucronatus* (seven PCGs); however, the start codon ATA is the most commonly used start codon in *P. vulnus* (six PCGs) and *X. americanum* (11 PCGs). Two complete stop codons (TAG and TAA) are used in the 12 PCGs of *M. graminicola*, which is also true for *P. vulnus* and most other non-plant parasitic nematodes [17], [33]. Some parasitic nematodes (including five other PPNs) also use a truncated stop codon (single T or TA) as a termination codon, as a complete canonical stop codon is created by posttranscriptional polyadenylation [34]. However, the phenomenon of the truncated stop codon was not found in *M. graminicola* (Table S2 in File S1).

**Figure 3:**
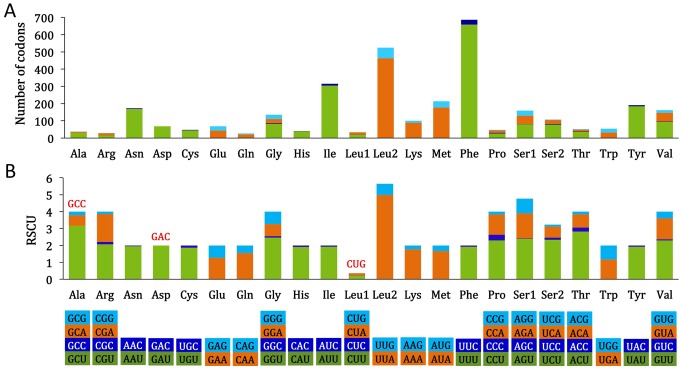
Codon usage pattern and relative synonymous codon usage (RSCU) of mtDNA of *Meloidogyne graminicola*. Numbers on the Y-axis refer to the total number of codons (A) and the RSCU value (B). Codon families are provided on the X-axis. Codons that are not present in the mitochondrial genome are indicated in red at the tops of the columns.

For the entire mtDNA sequence of *M. graminicola*, the nucleotide composition significantly favors A and T; this nucleotide bias is also reflected in codon usage (Figure 3). Excluding the stop codons, a total of 3,281 amino acids are encoded by the *M. graminicola* mt genome. Overall, the most frequently used amino acid is Phe (TTT), followed by Leu (TTA), Ile (ATT), Tyr (TAT), Met (ATA) and Asn (AAT). The proportion of these six amino acids is 59.8%, accounting for 1,963 amino acids; the codons encoding these amino acids are composed wholly of T and/or A, which may play an important role in the high A+T content of the entire mtDNA sequence. Three codons (GCC, GAC and CTG) were not observed in the PCGs. The absence of some codons was also reported for other PPNs [17], [25]. Analysis of the base composition at each codon position of the 12 PCGs showed that the third codon position (90.3%) is higher in A+T content than the first (80.2%) and second (79.7%) codon positions (Table S1 in File S1).

### Transfer RNA (tRNA) and ribosomal RNA genes

The secondary structure models of the tRNA genes in the mtDNA of *M. graminicola* were predicted using the tRNAscan-SE program [35]. All 22 mt tRNA genes of *M. graminicola*, ranging from 53 to 59 bp, lack a sequence encoding the typical cloverleaf tRNA secondary structure (Figure S2 in File S1). Out of 22 tRNA genes, 21 appear to encode tRNAs that lack a TΨC arm and loop, while the gene encoding *tRNA^Ser(AGN)^* lacks the sequence encoding a DHU loop. However, the majority of mitochondrial tRNA genes in nematodes have a unique predicted secondary structure in which the replaced TΨC arm is characterized by a loop of variable size (6–12 bases) [13], [17], [25], [26], [33]. In addition, *tRNA^Ser(UCN)^* of *M. graminicola* lacks the TΨC arm and loop, which was only found in a few nematodes such as *H. glycine* and *Ascaris suum* [29], [36]. All anticodons are conserved in nematodes, except that *tRNA^Arg^* uses the UCG anticodon, which deviates from the ACG anticodon used by most Chromadorean nematodes; the same phenomenon was reported in *R. similis* [25].

The *rrnS* and *rrnL* genes of *M. graminicola* were identified by sequence comparisons with those of other nematodes. Finally, the ends of two rRNA genes were assumed to extend to the boundaries of flanking genes according to the highest possibility of that PPN's alignment. The *rrnS* is located between *tRNA^Thr^* and *tRNA^Tyr^*, while *rrnL* is located between *tRNA^His^* and *nad3*. The sizes of the *rrnS* and *rrnL* genes are 596 and 815 bp, respectively, which are similar to those of other PPNs (Table 1). The A+T contents of *rrnS* and *rrnL* in *M. graminicola* are 84.23% and 87.24%, which are higher than those of other PPNs (Table S1 in File S1).

### Noncoding regions

The mtDNA of *M. graminicola* includes ten NCRs ranging from 3 to 5,063 bp. Of these, two larger NCRs (NCR1: 2,031 bp and NCR2: 5,063 bp) are located between *tRNA^Asp^* and *tRNA^Met^*, interrupted by *tRNA^Ser(UCN)^*, with A+T contents of 78.73% and 84.14%, respectively. The NCR in *M. graminicola* is obviously different from that of *M. javanica* because the latter has a ∼7 kb NCR and is not interrupted by any gene [30]. In NCR1, no repeat unit was identified. A remarkably large and nearly perfect 96-bp stem loop is located near *tRNA^Asp^* (Figure S3A in File S1), which has an A+T content of 89.58%. NCR2 is the largest AT-rich noncoding region, containing 25 repeated units of a 111-bp sequence and 3 repeated units of a 94-bp sequence with ten or six variants by base substitutions and/or indels respectively. The A+T contents are ∼84% in the 111-bp repeat unit and ∼95% in the 94-bp repeat unit. The 94-bp repeat unit is located at the end of NCR2 adjacent to *tRNA^Met^*. In contrast to *Caenorhabditis elegans* and *Ascaris suum*, no repeats of AT dinucleotides are found in the control region, which is often a conserved block in some insect mtDNAs [37], [38]. The 94-bp repeat unit and the truncated repeat may form a stable secondary structure (Figure S3B in File S1). In addition, two possible stem loops formed next to the repeat unit in NCR2, and, interestingly they have a similar stem loop structure (Figure S3C in File S1). Furthermore, no clear similarity in repeat unit(s) or secondary structures of NCRs were found between *M. graminicola* and *M. javanica*; the latter has three repeat units (8 bp, 63 bp and 102 bp) and two different stem loop structures [30]. In previous reports, these two repeat units (63 bp and 102 bp) seem relatively conserved among *M. javanica*, *M. incognita* and *M. arenaria* [30], but no similarity was identified in *M. hapla* [22].

The NCR of the mt genome in animals called the control region, which may act as the origin of replication and as a promoter for transcription initiation in animal mitochondrial DNA [39]. Inverted or direct repeats are often found in NCR and are considered to be the source of size variation in the entire mitochondrial genome. This length polymorphism may be useful for inferring the genetic structures of populations among closely related taxa and individuals of the same species due to the presence of a variable number of tandem repeat units [40]–[42].

In PPNs, NCRs range in size from 95 bp in *X. americanum* [24] to approximately 7 kb in *M. javanica* [30]. To date, the function of these NCRs remains unknown. In the mtDNA of *X. americanum*, no inverted or direct repeats were detected in the longest noncoding region (95 bp), but a conserved promoter motif may act as a bidirectional promoter for the transcription of both strands [24]. In *B. xylophilus* and *P. vulnus*, only a few different repeat units were identified in NCRs that would result in high A+T content or larger-sized mtDNA [17]. Among the five NCR subregions in *R. similis*, the fourth region, including the sequence along with a sextuple C folded into stable secondary structure, may play an important role in the origin of replication of the light strand [25].

While this revised article was in preparation, two papers were published online that reported three complete mt genomes: *M. graminicola* (20,030 bp) [43], *M. chitwoodi* (∼19.7 kb) and *M. incognita* (∼18.6–19.1 kb) [44]. The size of these mt genomes of *Meloidogyne* is well within the range detected in the completely sequenced Chromadoreans. But, the small difference was found in the two *M. graminicola* isolates (19, 589 bp in China isolate vs. 20,030 bp in Philippines isolate), this is likely due to difference in geographical populations. Comparing these four mt genomes, they shared the same PCGs and most tRNA genes arrangement, but there are three or four tRNAs locations different. Generally, the gene arrangement is highly conserved in interspecies within a genus. However, the translocation of *tRNA^Ile^* between the two species of *Metastrongylus* (*M*. *pudendotectus* and *M. salmi*) had been reported [45]. In addition, we found *tRNA^Val^* and *tRNA^Ser(UCN)^* are located between *tRNA^Asp^* and *atp6* in *M. graminicola* (Philippines isolate), while *tRNA^Val^* is located in a block of tRNA genes and *tRNA^Ser(UCN)^* is in the middle of two NCRs in *M. graminicola* (China isolate). The gene annotation of mt genome is problematic based on current tools, so the different algorithms to predict tRNAs could lead to different position of tRNA [46]. Here, we adopted the tRNAscan-SE program to predict tRNAs of *M. graminicola* (China isolate), this tool was widely used in the tRNAs detection of nematodes [16], [18], [24], [25], [29]. Furthermore, the slight difference existed in termination codons of *cox1* and *nad1* (TAA vs. T) between the two isolates of *M. graminicola*. The different geographical origin of nematodes may also result in the difference of the termination codon, the similar phenomenon has been reported in *B. xylophilus* [47]. In the NCR regions, it is obvious that the two isolates of *M. graminicola* showed the same types of tandem repeat units (111 bp and 94 bp), which do not match tandem repeat sequences in *M. chitwoodi* (48 bp, 92 bp and 111 bp) and *M. incognita* (8 bp, 63 bp and 102 bp) [44].

### Mitochondrial phylogeny of nematodes

**Figure 4:**
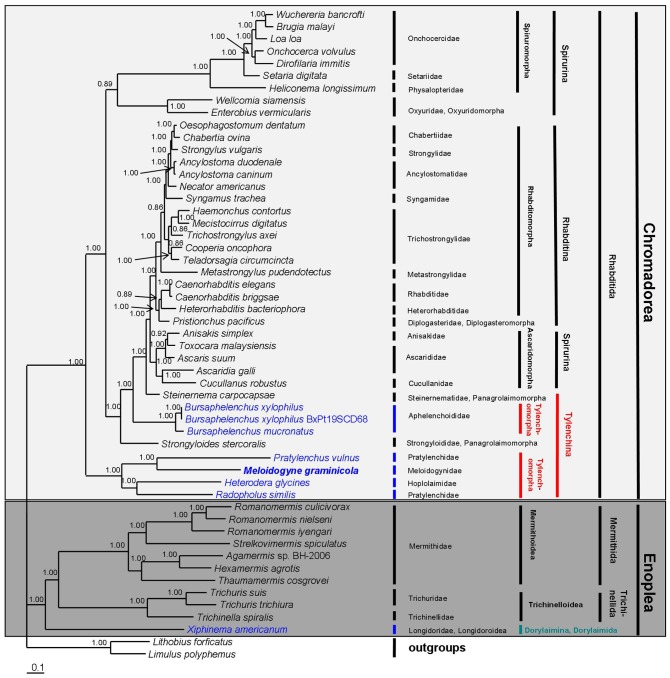
Phylogenetic tree from Bayesian analysis of amino sequences for 12 protein-coding genes for 50 nematode mitochondrial genomes. *Lithobius forficatus* and *Limulus polyphemus* were used as the outgroups. Numbers along the branches indicate Bayesian posterior probability (BPP) values. Classification according to De Ley and Blaxter [6].

**Figure 5:**
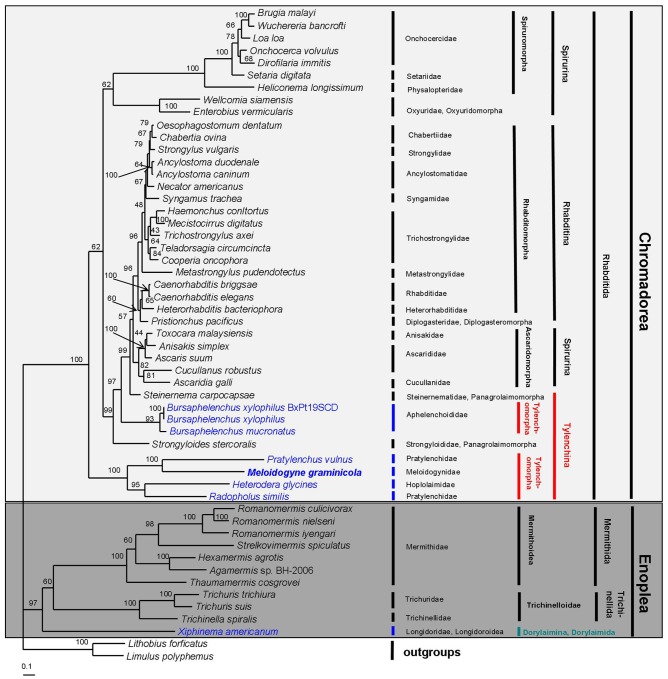
Phylogenetic tree from maximum likelihood analysis of amino sequences for 12 protein-coding genes for 50 nematode mitochondrial genomes. *Lithobius forficatus* and *Limulus polyphemus* were used as the outgroups. Bootstrap percentage (BP) values are indicated at the nodes. Classification according to De Ley and Blaxter [6].

We constructed phylogenetic trees to examine the relationships between 50 species of nematodes, including the newly sequenced *M. graminicola*, using two different methods (Bayesian inference [BI] and maximum likelihood [ML]) employing different building strategies and/or different distance models based on the combined amino acid sequences of 12 protein-encoding genes (∼2,176 amino acids each). The topologies of the Bayesian tree (Figure 4) and ML tree (Figure 5) are identical, with only small differences in Bayesian posterior probability (BPP) and bootstrap percentage (BP) of ML analyses. The two dendrograms are similar to the phylogenetic trees constructed based on previously reported mt genome analysis [17], [18]. When two Arthropod species, i.e., *Lithobius forficatus* and *Limulus polyphemus*, were used as outgroup taxa, the two resulting trees both contained two main clades that belong to the class Enoplea and the class Chromadorea, respectively.

11 species including the PPN *X. americanum* (from the class Enoplea) form a strongly supported monophyletic clade (1.00 BPP in BI and 97% BP in ML) of Enoplea. Of the 11 species, 7 species form a monophyletic clade belonging to the order Mermithida with high support (1.00 BPP and 100% BP), other 3 species form a monophyly of the order Trichinellida with high support (1.00 BPP and 100% BP), and the remaining one species (*X. americanum*) belonging to the order Dorylaimida occupy a basal position within a highly supported clade (1.00 BPP and 97% BP) with Mermithida and Trichinellida. Among the three orders, the Mermithida is sister to the Trichinellida with strong support in the Bayesian tree (1.00 BPP), but weak support in ML tree (60% BP).

The 39 remaining species from the class Chromadorea form a monophyletic group belonging to the order Rhabditida with high support (1.00 BPP in BI and 100% BP in ML). The monophyly contains three suborders, namely Rhabditina (17 species), Spirurina (14 species) and Tylenchina (8 species). Among the three suborders, the monophyletic clade of the Rhabditina is highly supported (1.00 BPP in BI and 96% BP in ML), but the Spirurina and the Tylenchina are both paraphyletic. In the clade of Rhabditina, there are two infraorders: Rhabditomorpha (16 species) and Diplogasteromorpha (1 species). Among these, 13 species of the Rhabditomorpha form a monophyletic clade with strong support (1.00 BPP in BI and 96% BP in ML), the other 3 species of the Rhabditomorpha and one species of the Diplogasteromorpha are positioned in the same cluster with high support in the BI tree (1.00 BPP), however weak support in the ML tree (60% BP). The Spirurina includes three infraorders: Spiruromorpha (7 species), Oxyuridomorpha (2 species) and Ascaridomorpha (5 species). The three infraorders are all monophyletic with high support (1.00 BPP in BI and 100% BP in ML) except the clade of Ascaridomorpha in ML analysis with moderate support (82% BP). The Ascaridomorpha is closely related to the Rhabditina. In addition, the Spiruromorpha and the Oxyuridomorpha formed a monophyly that is far away from the Ascaridomorpha. The Tylenchina contains two infraorders, Tylenchomorpha (6 species) and Panagrolaimomorpha (2 species), and they are both not monophyletic. In this study, our main aim was to assess the phylogenetic relationships between the Tylenchomorpha, especially to infer the phylogenetic position of *Meloidogyne* in relation to other Tylenchomorpha. The six infraorder Tylenchomorpha species from two superfamilies (Aphelenchoidea and Tylenchoidea) were paraphyletic within the clade of Chromadorea in both trees. *B. xylophilus* and *B. mucronatus* of Aphelenchoidea are positioned in the cluster comprising Panagrolaimomorpha, Ascaridomorpha, Diplogasteromorpha and Rhabditomorpha and are closely related to the two Panagrolaimomorpha species (*Strongyloides stercoralis* and *Steinernema carpocapsae*), with high support (1.00 BPP and >97% BP). The other four species of Tylenchoidea, including *M. graminicola*, *P*. *vulnus*, *H. glycines* and *R. similis*, were placed in a separate clade far from *B. xylophilus* and *B. mucronatus*. These results indicate that the Tylenchomorpha is not monophyletic, which is consistent with previously reported mitochondrial and SSU phylogenies [17], [18], [48].

All four Tylenchoidea species (including *M. graminicola*) reside within a well-supported monophyletic clade in our BI and ML trees (1.00 BPP and 100% BP). In the clade of Tylenchoidea, *M. graminicola* is sister to *P*. *vulnus*, with very strong support (1.00 BPP in BI and 100% BP in ML), and *H. glycines* is sister to *R. similis*, with high support (1.00 BPP and 95% BP). This phylogenetic analysis revealed that *Meloidogyne* has a closer relationship to *Pratylenchus* (within Pratylenchidae) than to *Heterodera*. De Ley and Blaxter [6] considered it appropriate to classify Meloidogyninae as a fully separate family and to include Heteroderinae as a subfamily within Hoplolaimidae based on molecular and morphological data. Subsequently, several SSU- and LSU-based phylogenetic studies have indicated that RKN is closely related to Pratylenchidae, whereas CN is closely related to Hoplolaimidae [3], [8]–[10]. For example, in the SSU analysis of Bert *et al*. [3], RKN and three genera of the family Pratylenchidae including *Pratylenchus*, *Zygotylenchus* and *Hirschmanniella* were grouped in a highly supported clade, whereas CN (*Heterodera* and *Globodera*) and Hoplolaimids (*Rotylenchus*, *Scutellonema*, *Rotylenchulus* and *Helicotylenchus*) were within the same clade with high support; in the phylogenetic trees inferred from SSU described by Holterman *et al*. [9], [10], RKN were placed within the Pratylenchidae, and CN and the Hoplolaimidae were closely related sister families; in the BI trees based on LSU D2D3 inferred from Subbotin *et al*. [8], RKN, *Pratylenchus* and *Hirschmanniella* grouped in a highly supported clade, More recently, Rybarczyk-Mydłowska *et al*. [11] proposed that RKN arose from Pratylenchidae, not from one of the economically high-impact lesion nematodes, based on SSU and RNA polymerase II data. Close relationships between RKN and Pratylenchidae and between CN and Hoplolaimidae were already postulated based on their similar head end-on views [49]. The morphological similarity between RKN and Pratylenchidae in combination with previous rDNA phylogeny and the current mt genome findings strongly suggest a close relationship between RKN and Pratylenchidae, and an independent family of RKN, i.e., Meloidogynidae.

The current phylogenetic analysis of the mt genome show that *R. similis* and *P. vulnus*, the two members of the family Pratylenchidae are not sister. *R. similis* is instead sister to *H. glycines* of Hoplolaimidae (or Heteroderidae). Due to the presence of some similar morphological characters and feeding modes between the burrowing nematodes and some members of Pratylenchidae, the traditional placement of *R. similis* in the family Pratylenchidae was universally accepted. However, several recent SSU and LSU phylogeny analyses have shown that *R. similis* is sister to the Hoplolaimidae or Heteroderidae [3], [8]–[11]. Subbotin *et al*. [8] rejected the hypotheses placing *Radopholus* within the family Pratylenchidae because *Radopholus* clusters with Hoplolaimidae or Heteroderidae in their LSU phylogenetic trees. Morphological similarities shared between *Radopholus* and some of the Hoplolaimidae, including the presence of protrusible gubernaculums and secondary sexual dimorphism that differs from the Pratylenchidae, have been noted [5], [9]. Thus, taking morphology, previous rDNA and current mt genome phylogenetic analyses into account, the classification of *Radopholus* should be revised.

## Materials and Methods

### Ethics statement

No specific permissions were required for the nematode collected for this study in Hainan Province, China. The field for nematodes collection was neither privately owned nor protected, and did not involve endangered or protected species.

### Nematode collection and DNA Extraction

The root-knot nematode *Meloidogyne graminicola* was isolated from infected rice (Wenchang, Hainan Province, China) and maintained in the greenhouse. The species was first morphologically identified, and its identity was confirmed by isozyme and molecular data obtained in the laboratory (Figure S4–S6 in File S1; Table S3 in File S1). *Meloidogyne* populations were purified from single egg masses and reared on rice (Guinongzhan).

Total genomic DNA was extracted from pooled nematodes using the following method [50]: Approximately 50 µl of fresh second-stage juveniles (J2s) were ground in a mortar and pestle with liquid nitrogen until the nematodes were homogenized. Then, 500 µl of extraction buffer (200 mM NaCl, 200 mM Tris pH 7.5, 20 mM EDTA, 2% SDS, 0.04 M 2-mercaptoethanol, 0.2 mg/ml proteinase K) was added to the homogenate. The homogenate was incubated at 40°C for 30 minutes, followed by extraction with phenol:chloroform and chloroform. DNA was precipitated with two volumes of ethanol and 0.1 volume of 3 M sodium acetate pH 5.2, washed in 70% ethanol and resuspended in TE (10 mM Tris pH 8.0, 1 mM EDTA). No additional purification was required for subsequent procedures. This DNA, which was made suitable for use in PCR by diluting to 10 ng/µl in ddH2O, was stored at −20°C until use.

### Mitochondrial genome amplification and cloning

Initially, two partial fragments from *cox1* and the *cox2*-*cob* gene region were amplified using the primer sets COIF/COIR [24] and C2F3/MMT2 [19], [51], respectively (Table S4 in File S1). PCR was carried out in a 50 µl reaction volume containing 1× buffer, 1 U KOD-FX (TOYOBO, Shanghai, China), 0.4 mM dNTP, 0.3 µM primers and ∼10 ng of total DNA. The cycling conditions were 94°C for 2 min, 35 cycles at 98°C for 10 s, 50°C for 30 s and 68°C for 2 min, followed by an extension at 68°C for 5 min. Two PCR fragments were cloned into the *pMD18*-*T* vector (TaKaRa, Dalian, China). Subsequently, two additional primer sets COILF1/16SLR1 and COIIF2/COIR2 (Table S4 in File S1) were then designed for long PCR amplification based on the nucleotide sequences of the above two fragments. First, an approximately 6-kb fragment of the *M. graminicola* mitochondrial genome was amplified using the primer sets COILF1/16SLR1 under normal long PCR conditions (described above, except for a 7-min extension in each cycle). Second, the primer sets COIIF2/COIR2 and the following PCR conditions were used to amplify an additional larger fragment (∼15 kb): PCR was performed with a TaKaRa PCR Thermal Cycler Dice Gradient TP600 (TaKaRa, Dalian, China) in a 50-µl volume. The cycling conditions were as follows: 94°C for 2 min (initial denaturation), 35 cycles at 98°C for 10 s, 50°C for 30 s (annealing) and 62°C (extension) for 15 min, and a final extension at 62°C for 7 min. Both products were excised from a gel and purified using a gel purification kit (Axygen Biotechnology, Hangzhou, China) and cloned into plasmid *pJAZZ-OK* (Lucigen, USA) according to the manufacturer's instructions. Cycle sequencing reactions were done using the BigDye Terminator v3.1 chemistry on an ABI 3730 xl DNA analyzer at Taihe Biotechnology Company (Beijing, China) using a primer walking strategy. For noncoding region, a series of recombinant subclones, which cover the noncoding region of the long PCR fragment, were isolated by screening the transformed library; the sequences of overlapping fragments were double-checked and then assembled to obtain the complete strand of the entire genome.

### Sequence assembly and mitochondrial genome annotation

Nucleotide sequences were assembled and analyzed using SeqMan version 7 (DNAStar Lasergene, USA). The open-reading frames and codon usage profiles of 12 mitochondrial protein-coding genes (PCGs) were analyzed with the Open Reading Frame Finder (http://www.ncbi.nlm.nih.gov/gorf/gorf.html) or the DNA-to-protein translation web-service (http://insilico.ehu.es/translate/) using the invertebrate mitochondrial genetic code, and subsequently, the initiation and termination codons of each PCGs were determined by comparing the inferred amino acid sequences with those of other nematode species reported previously.

The online tRNAscan-SE service (http://selab.janelia.org/tRNAscan-SE/) [35] was used to search for tRNA genes with lower cut-off values (cove score cutoff  = 13) because this strategy has been used for the detection of a range of insect and cyst nematode tRNA genes [29]. Moreover, an additional criterion was used to identify tRNA genes [29]: tRNA genes should not be located within a protein-coding gene, but they should lie in the small intergenic regions between genes (or in the longer noncoding regions). The 12 S and 16 S rRNA genes were identified by BLAST searches or by comparing the sequences of these genes with those of other plant parasitic nematodes. Tandem repeats in the noncoding regions were found using the Tandem Repeat Finder program (http://tandem.bu.edu/trf/trf.html) [52].

### Phylogenetic analyses

Considering the high degree of interspecific variation in nucleotide sequences of mitochondrial genes of nematodes [33], the deduced amino acid sequences of mitochondrial proteins were used for phylogenetic analyses. All species used in phylogenetic analyses are listed in Table S5 in File S1. Two arthropods (*Lithobius forficatus* and *Limulus polyphemus*) were used as the outgroup according to a previous report [17]. The derived amino acid sequences of 12 mitochondrial PCGs from 50 nematode species were aligned individually using the online MAFFT service with default parameters (http://mafft.cbrc.jp/alignment/server/) [53] and were then concatenated into a single alignment using SequenceMatrix [54]. Ambiguous sites and regions in the alignment were excluded using Gblocks 0.91 b (http://www.phylogeny.fr/version2_cgi/one_task.cgi?task_type=gblocks ) [55] under less stringent selection, allowing gap positions within the final blocks and less strict flanking positions.

Phylogenetic reconstructions were performed using MrBayes3.2.2 [56] and PhyML3.1 [57]. ProtTest 3.3 [58] selected the MtArt [59] model, with a proportion of invariable sites (+I), a gamma distribution (+G) and empirical base frequencies (+F) as the best-fit substitution model based on the Akaike information criterion (AIC) [60], followed by LG [61] and WAG [62], again using the options +I, +G and +F.

Since neither the MtArt model nor the LG model is implemented in MrBayes, WAG, the next best available model, was used for the edited dataset (with parameter +I, +G and +F). Therefore, both MrBayes and PhyML analyses were run with the WAG+I+G+F model. MrBayes analyses were performed using two parallel runs with four chains each, which were run for 1,500,000 metropolis-coupled MCMC generations, sampling a tree every 100 generations. The first 3,750 trees (25%) represented burn-in, and the remaining trees were used to calculate Bayesian posterior probabilities (BPP). Maximum likelihood (ML) analysis was performed using PhyML3.1 with the alternative WAG model. A total of 100 bootstrap replicates were run and bootstrap percentages (BP) were calculated. Phylograms were drawn using FigTree v.1.31 (http://tree.bio.ed.ac.uk/software/figtree/).

## Supporting Information

File S1 **This supporting information file contains Figures S1–S6 and Tables S1–S5.** Figure S1: Mitochondrial gene arrangement of representative nematodes. Gene and genome size are not scale. Noncoding region were not shown. Red lines below the gene order map indicate genes are encoded by the other strand. Figure S2: Predicted secondary structures of 22 tRNAs of *Meloidogyne graminicola*. Figure S3: Predicted stem–loop structures of two noncoding regions. **A**) Noncoding region of the 96-bp sequence near *tRNA^Asp^* in NCR1. **B**) Noncoding region of the 94-bp sequence and the truncated 34-bp sequence in the end of NCR2. **C**) Noncoding regions (73 bp and 82 bp) next to the 111-bp repeat unit in NCR2. Figure S4: Light micrograph of a perineal pattern of a female of *Meloidogyne graminicola*. Figure S5: Esterase (EST) and malate dehydrogenease (MDH) phenotypes in *Meloidogyne graminicola*. Figure S6: PCR product (left) by using primer pairs C2F3/1108 and the corresponding sequence (right) of *cox2*-*tRNA^His^*-*rrnL* of *Meloidogyne graminicola*.
(PDF)
