# Supplementary material for: The Complete Mitochondrial Genome of Meloidogyne graminicola (Tylenchina): A Unique Gene Arrangement and Its Phylogenetic Implications
Source: PLoS One. 2014 Jun 3;9(6):e98558. doi: 10.1371/journal.pone.0098558 (PMC4043755; doi:10.1371/journal.pone.0098558)
Supplement: File S1 — This supporting information file contains Figures S1–S6 and Tables S1–S5. Figure S1: Mitochondrial gene arrangement of representative nematodes. Gene and genome size are not scale. Noncoding region were not shown. Red lines below the gene order map indicate genes are encoded by the other strand. Figure S2: Predicted secondary structures of 22 tRNAs of Meloidogyne graminicola. Figure S3: Predicted stem–loop structures of two noncoding regions. A) Noncoding region of the 96-bp sequence near tRNAAsp in NCR1. B) Noncoding region of the 94-bp sequence and the truncated 34-bp sequence in the end of NCR2. C) Noncoding regions (73 bp and 82 bp) next to the 111-bp repeat unit in NCR2. Figure S4: Light micrograph of a perineal pattern of a female of Meloidogyne graminicola. Figure S5: Esterase (EST) and malate dehydrogenease (MDH) phenotypes in Meloidogyne graminicola. Figure S6: PCR product (left) by using primer pairs C2F3/1108 and the corresponding sequence (right) of cox2-tRNAHis-rrnL of Meloidogyne graminicola. (PDF) [file pone.0098558.s001.pdf]

Supporting Information File S1

The complete mitochondrial genome of *Meloidogyne graminicola* (Tylenchina): a unique gene arrangement and its phylogenetic implications

Longhua Sun[1,2], Kan Zhuo[1,2*], Borong Lin[1,2], Honghong Wang[1,2], Jinling Liao[1,2*]

1 Laboratory of Plant Nematology, South China Agricultural University, Guangzhou 510642, China, **2** Guangdong Province Key Laboratory of Microbial Signals and Disease Control, South China Agricultural University, Guangzhou 510642, China

Figure S1. Mitochondrial gene arrangement of representative nematodes. Gene and genome size are not scale. Noncoding region were not shown. Red lines below the gene order map indicate genes are encoded by the other strand.

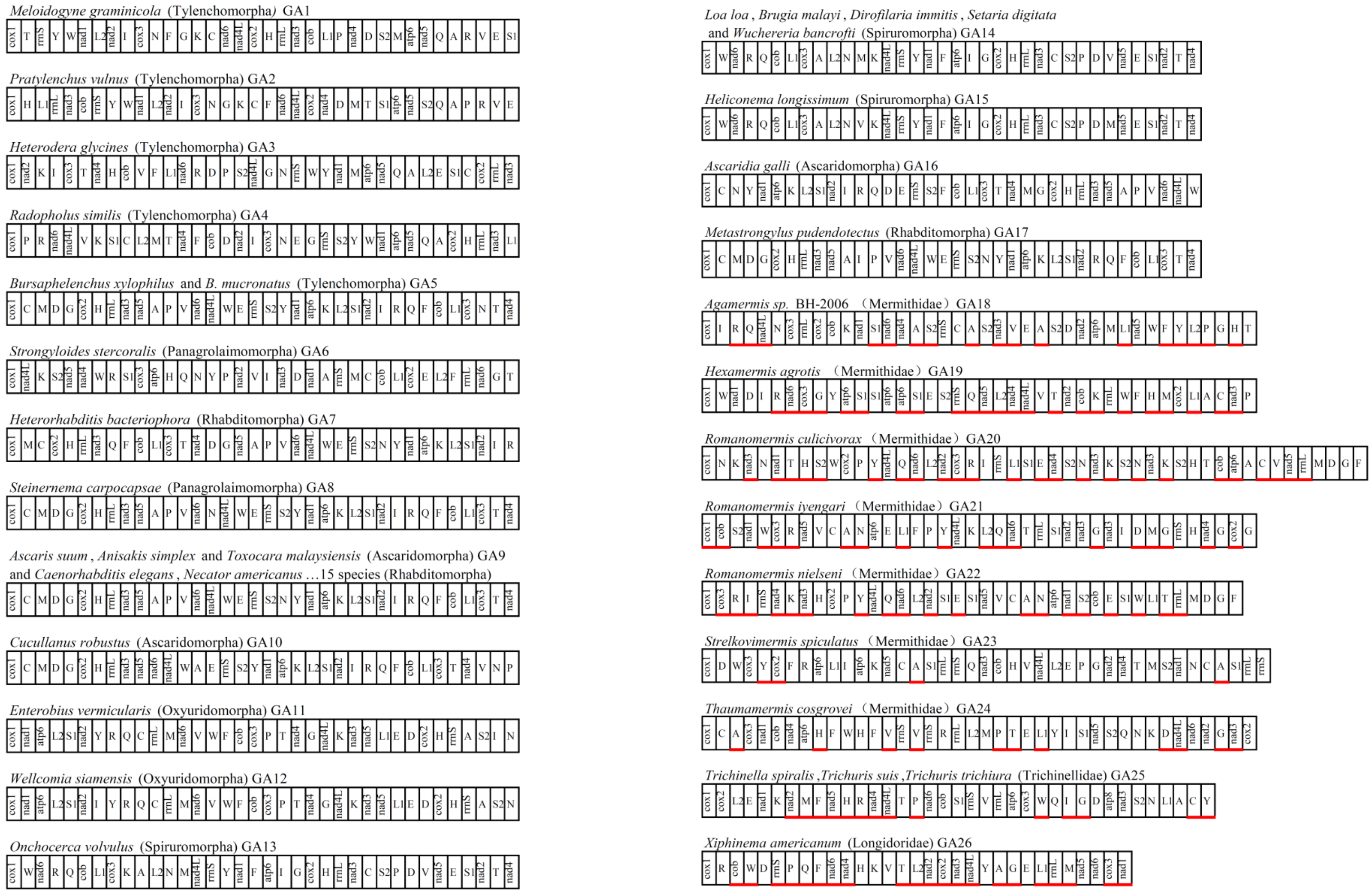

Figure S2. Predicted secondary structures of 22 tRNAs of *Meloidogyne graminicola*.

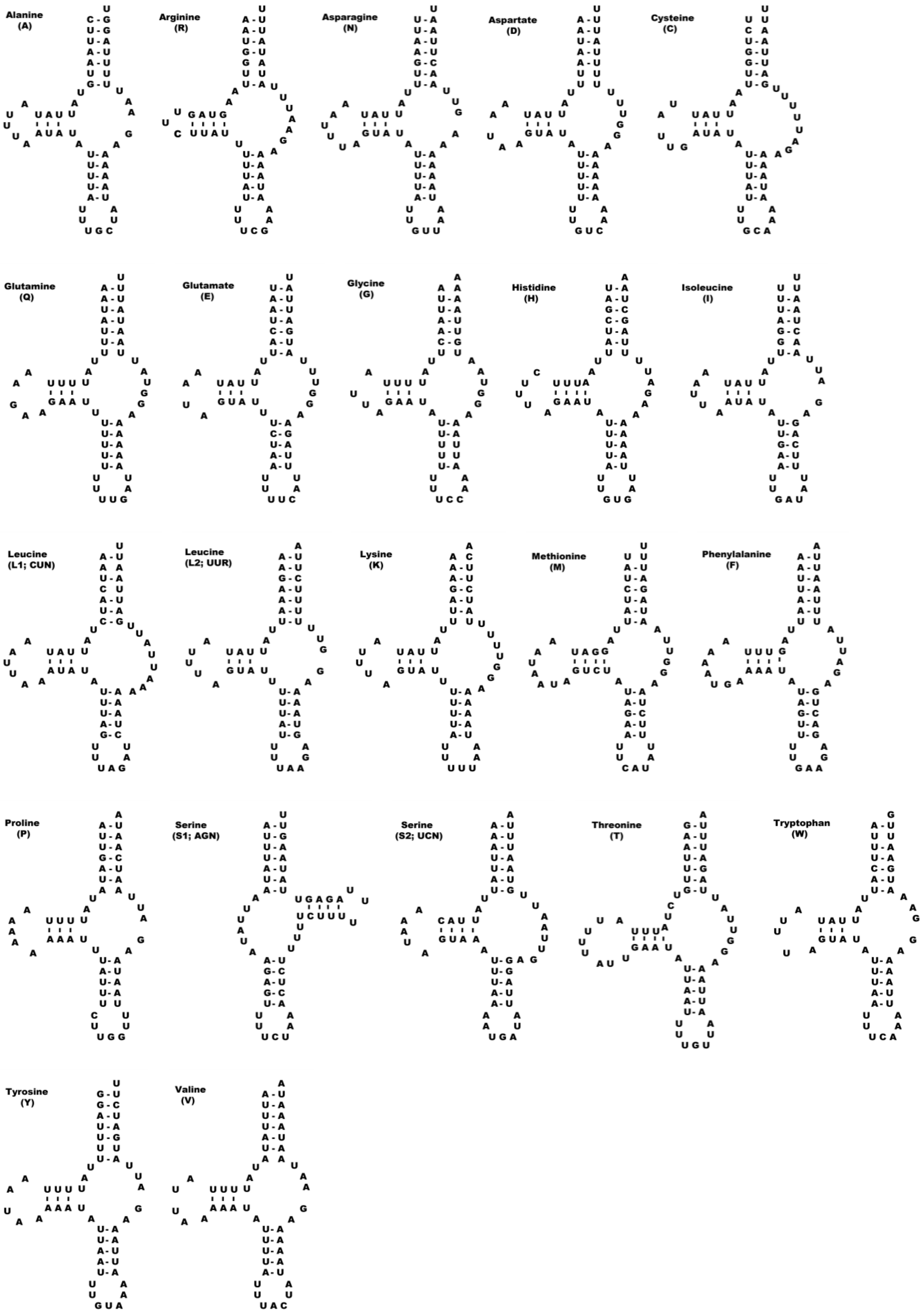

Figure S3. Predicted stem–loop structures of two noncoding regions. A) Noncoding region of the 96-bp sequence near *tRNA^{Asp}* in NCR1. **B**) Noncoding region of the 94-bp sequence and the truncated 34-bp sequence in the end of NCR2. **C**) Noncoding regions (73 bp and 82 bp) next to the 111-bp repeat unit in NCR2.

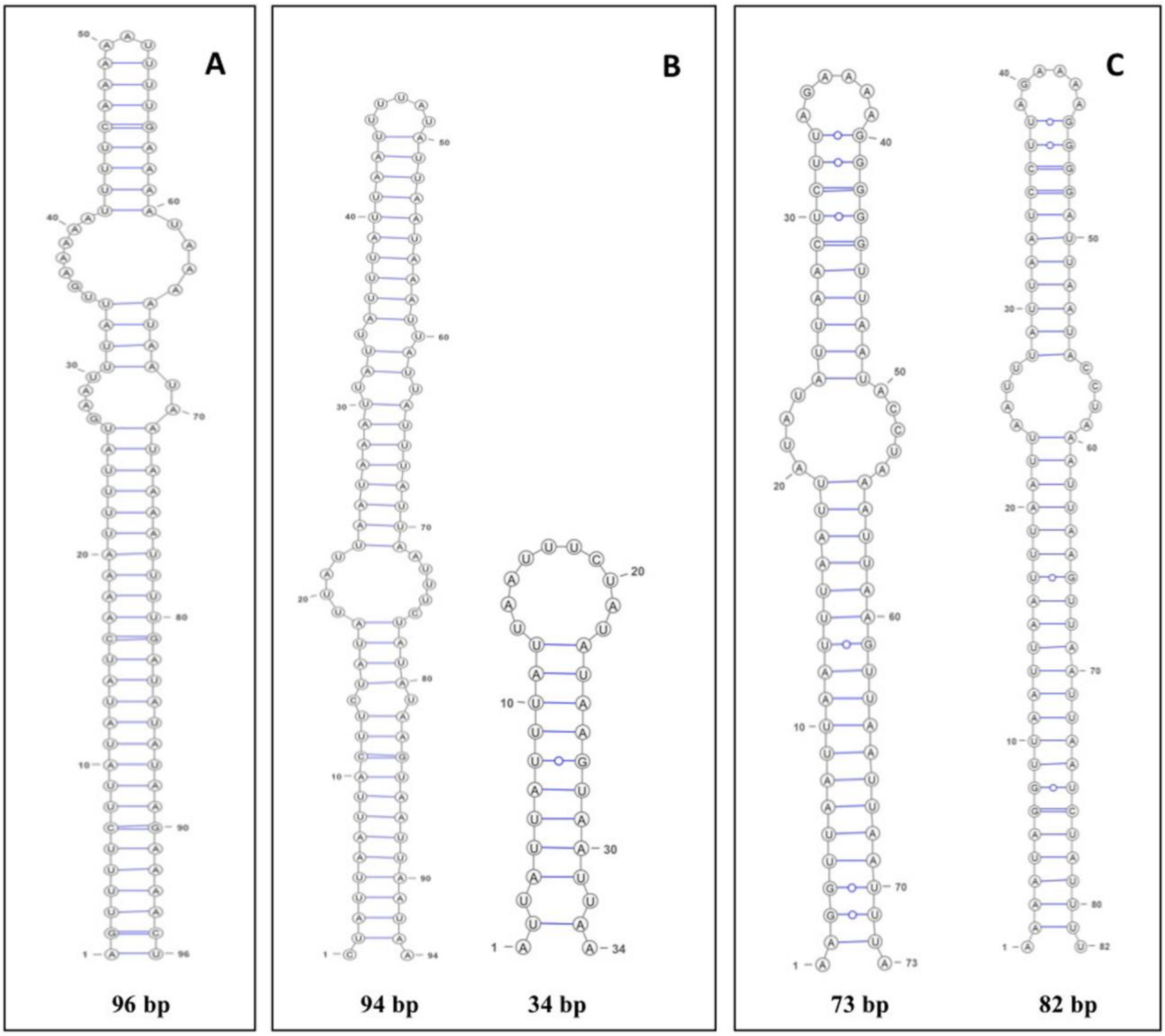

Figure S4. Light micrograph of a perineal pattern of a female of *Meloidogyne graminicola*.

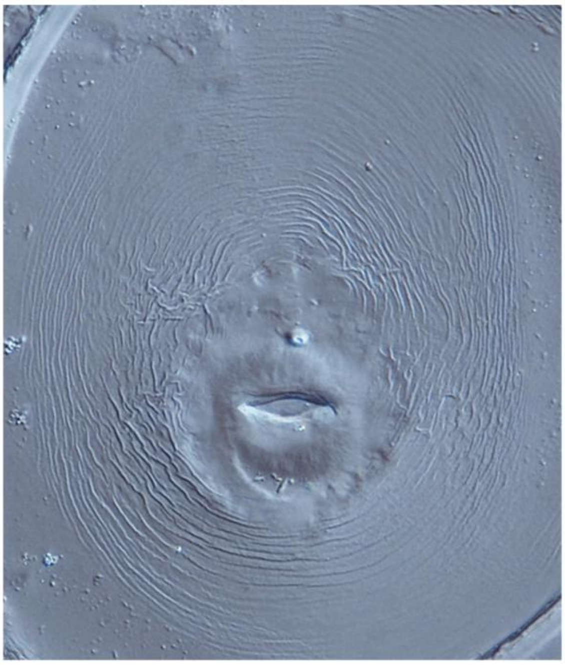

Figure S5. Esterase (EST) and malate dehydrogenease (MDH) phenotypes in *Meloidogyne graminicola*. Left: EST (VS1); right: MDH (N1a) Mj: *M. javanica* (control); Mg: *M. graminicola*

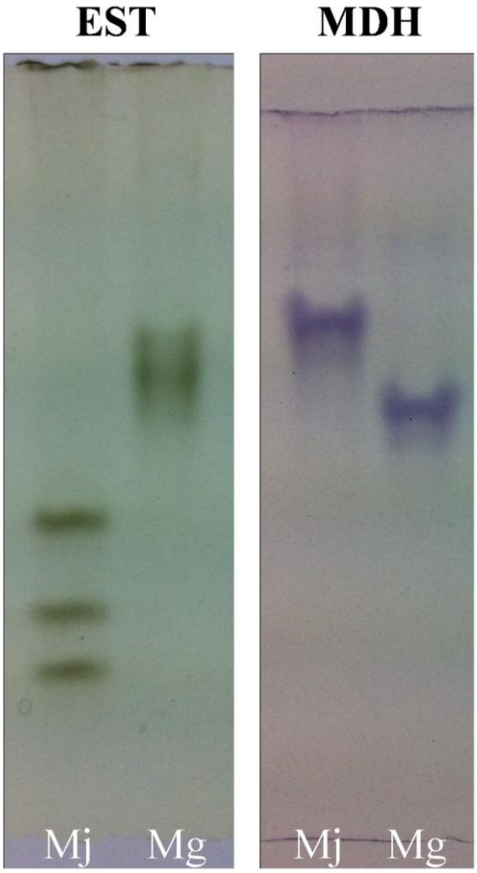

Figure S6. PCR product (left) by using primer pairs C2F3/1108 and the corresponding sequence (right) of *cox2-tRNA*^{His}*-rrnL* of *Meloidogyne graminicola*.

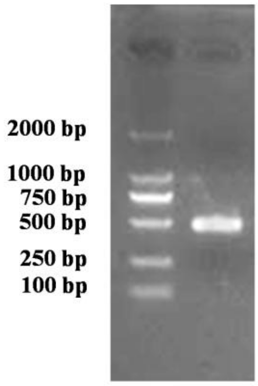


```
  1 GGCAAATCAT TCTTTTATAC CTATTGTTTT AGAAATTGTT TTATTTGATT
 51 TTTTTAAGTT AATTTTGATT AGCTATTAAT TTCTTTAGAA TATTTTATTG
101 TGATTAAAAA AGATTTTAGC TAATGTTATT TTTTAAATAT TTTTTATTGT
151 TGAAGAAATT AGGATATATT TATATATTTA TAATAATTAA AATTTTAAAA
201 AATTTTTTAT TTGATTTTTA ATTTTATATA AAAATTATAA TTATTAATAT
251 ATAGAAATTT TGATTAAAAA GATTTTAATT TTTTTGTTAT ATTTTTTATT
301 ATAATATTTT TTTTTTTTAA TGTTAATTTT CATTAAATAA TAATTTAATT
351 ATTATTATTC TATTTATTAT TTATTATTAA ATTAATAATA AGTTTGAATT
401 AGATTTAATA TAAAATATTT CTTAAAATTT GAGAAAATTT AGGTTTTGAT
451 TTTTTATTTC TACTCGATGA AAATTAAATT GTATCTTT
```

Table S1. Comparison of A + T content, AT- and GC-skew of the protein-coding genes, rRNA genes and noncoding regions of mitochondrial genomes of the plant parasitic nematodes sequenced to date.

	**AT content**							**AT-skew**							**GC-skew**						
	**MG**	**PV**	**HG**	**RS**	**BX**	**BM**	**XA**	**MG**	**PV**	**HG**	**RS**	**BX**	**BM**	**XA**	**MG**	**PV**	**HG**	**RS**	**BX**	**BM**	**XA**
atp6	86.78	75.26	85.26	87.50	78.45	83.08	67.80	-0.356	-0.311	-0.593	-0.272	-0.300	-0.278	-0.356	0.605	0.403	0.142	0.440	0.312	0.347	-0.106
cox1	75.36	68.10	75.13	77.26	72.62	75.82	64.60	-0.295	-0.230	-0.371	-0.232	-0.218	-0.241	-0.295	0.326	0.309	0.172	0.266	0.262	0.228	0.118
cox2	80.74	76.65	77.55	82.60	77.68	77.97	63.12	-0.281	-0.218	-0.419	-0.221	-0.246	-0.204	-0.281	0.415	0.371	0.126	0.390	0.390	0.276	0.188
cox3	81.45	72.20	80.45	82.04	76.04	78.39	64.13	-0.350	-0.283	-0.471	-0.320	-0.353	-0.302	-0.350	0.356	0.333	0.127	0.367	0.326	0.277	0.086
cob	81.35	71.45	80.67	82.97	77.57	81.29	65.94	-0.333	-0.211	-0.479	-0.325	-0.286	-0.285	-0.333	0.285	0.266	0.063	0.438	0.352	0.330	0.051
nad1	84.29	77.09	82.32	83.74	78.69	80.02	66.67	-0.346	-0.254	-0.567	-0.348	-0.342	-0.338	-0.346	0.454	0.210	0.092	0.400	0.269	0.200	0.090
nad2	90.45	80.05	88.62	88.97	89.21	91.55	71.21	-0.340	-0.243	-0.534	-0.327	-0.378	-0.372	-0.340	0.558	0.256	0.181	0.670	0.371	0.257	0.137
nad3	87.59	77.88	89.52	90.69	87.58	86.04	72.01	-0.269	-0.346	-0.545	-0.298	-0.370	-0.370	-0.269	0.737	0.507	0.086	0.677	0.805	0.379	0.326
nad4	83.93	76.65	82.98	86.62	83.66	86.10	66.67	-0.352	-0.367	-0.504	-0.355	-0.361	-0.337	-0.352	0.340	0.286	0.245	0.497	0.294	0.275	0.199
nad4L	86.58	81.82	86.67	91.36	87.18	89.32	62.22	-0.362	-0.513	-0.313	-0.288	-0.422	-0.407	-0.362	0.393	0.429	-0.067	0.619	0.667	0.680	0.353
nad5	86.03	77.89	86.50	87.37	83.94	86.04	67.64	-0.375	-0.301	-0.563	-0.326	-0.368	-0.370	-0.375	0.476	0.245	0.222	0.371	0.468	0.379	-0.066
nad6	90.51	79.72	84.28	91.37	88.51	89.20	70.29	-0.360	-0.421	-0.595	-0.197	-0.361	-0.423	-0.360	0.351	0.471	-0.015	0.389	0.600	0.745	0.008
rrnS	84.23	66.62	84.85	84.10	83.28	85.34	61.86	-0.116	-0.068	-0.222	-0.093	-0.060	-0.069	-0.116	0.277	0.223	0.333	0.327	0.248	0.360	-0.106
rrnL	87.24	71.51	86.23	85.84	86.71	88.24	69.68	-0.159	-0.172	-0.309	-0.090	-0.100	-0.095	-0.159	0.500	0.271	0.351	0.362	0.381	0.429	-0.113
PCGs-1st	80.20	70.60	77.20	91.70	75.30	81.10	64.10	-0.229	-0.218	-0.399	-0.254	-0.243	-0.223	-0.229	0.455	0.388	0.237	0.679	0.466	0.439	0.173
PCGs-2nd	79.70	70.10	79.60	82.00	74.90	75.00	64.50	-0.486	-0.478	-0.593	-0.239	-0.487	-0.443	-0.486	0.275	0.164	0.059	0.489	0.171	0.208	-0.020
PCGs-3rd	90.30	84.70	90.50	50.20	91.20	92.50	71.10	-0.307	-0.188	-0.507	-0.064	-0.265	-0.308	-0.307	0.531	0.425	0.104	0.202	0.563	0.307	0.118
tRNA	87.11	77.14	85.91	84.84	84.70	85.60	64.72	-0.111	-0.094	-0.215	-0.156	-0.077	-0.051	-0.111	0.455	0.395	0.398	0.388	0.364	0.375	-0.076
NCR*	82.59	72.90	0.00	87.28	98.70	99.24	71.88*	-0.176	0.026	0.000	-0.171	-0.033	0.002	-0.176*	0.186	0.166	0.000	0.396	0.000	-0.263	-0.333*
Genome	83.51	73.90	82.65	85.40	83.50	85.30	66.52	-0.250	-0.139	-0.415	-0.239	-0.238	-0.233	-0.250	0.318	0.249	0.199	0.394	0.345	0.320	-0.075

Abbreviations: MG: *Meloidogyne graminicola*; PV: *Pratylenchus vulnus*; HG: *Heterodera glycine*; RS: *Radopholus similis*; BX: *Bursaphelenchus xylophilus*; BM: *B. mucronatus*; XA: *Xiphinema americanum*;

[a]: GC- and AT-skew is calculated as (A%-T%)/(A%+T%) and (G%-C%)/(C%+G%)

*: Data from the longest noncoding region (95 bp) in *X. americanum*.

NA: Noncoding region (NCR) data are not available for *H. glycines*.

Table S2. Properties of protein-coding genes and two ribosomal RNA genes of the mitochondrial genomes of plant parasitic nematodes sequenced to data.

Gene/Regions	No. of amino acids (AA) /nucleotides (bp)							Inferred initiation codon/ Inferred termination codon						
	**MG**	**PV**	**HG**	**RS**	**BX**	**BM**	**XA**	**MG**	**PV**	**HG**	**RS**	**BX**	**BM**	**XA**
atp6	178	193	189	199	198	198	205*	TTT/TAA	ATT/TAG	ATT/TAG	ATT/TAG	ATT/T	ATA/TAG	ATA/T(AA)
cox1	517	510	507	519	520	520	518	ATT/TAA	ATA/TAA	GTT/TAA	ATA/?	ATT/TAA	ATT/TAA	ATA/T(AA)
cox2	224	226	218	226	229	229	206*	ATT/TAA	ATA/TAG	ATT/TAA	TTA/?	ATT/TAG	ATT/TAG	ATA/TA(A)
cox3	256	258	253	257	255	255	249*	TTG/TAA	ATA/TAG	TTT/TAA	TTG/TAG	ATT/TAA	ATT/TAA	ATA/TAA
cob	344	368	357	362	367	367	366*	ATA/TAG	ATA/TAG	TTC/?	ATA/?	ATA/T	ATA/T	ATA/TAA
nad1	294	290	277	286	290	291	289*	TTG/TAA	TTG/TAA	GTT/TAA	ATA/TAG	ATT/TAA	ATA/TAA	ATA/TAA
nad2	268	273	267	275	274	275	278*	ATT/TAA	ATA/TAG	ATT/TAA	ATG/?	ATT/TAA	ATT/TAA	ATA/T(AA)
nad3	101	109	111	111	109	109	106*	ATT/TAG	ATT/TAG	ATT/T	ATA/?	ATT/TAA	ATA/TAA	ATA/T(AG)
nad4	389	403	383	406	409	409	394*	ATA/TAG	ATA/TAA	ATT/TAA	TTG/?	ATA/TAA	ATT/TAA	ATG/TAA
nad4L	81	76	74	80	77	77	91*	ATT/TAA	ATT/TAG	ATT/TAA	ATT/TAG	ATA/TAA	ATT/TAA	ATA/TAA
nad5	500	486	512	511	522	522	515	ATA/TAA	ATT/TAA	ATT/T	TTA/TAG	ATT/TAA	ATT/TAA	ATA/TAA
nad6	129	142	146	139	144	144	146	ATT/TAG	TTG/TAG	ATT/T	ATA/?	ATT/TAA	TTG/TAA	ATA/TAA
rRNA genes														
rrnS	596	686	673	692	700	682	569*							
rrnL	815	895	806	840	948	952	729*							
EmtG (AA)	3,281	3,334	3,295	3,371	3,394	3,396	3,363							
EmtG (bp)	19,589	21,656	14,915	16,791	14,778	14,583	12,626							

Results based on GenBank accessions, publications and alignment analyses.

a: partial mitochondrial genome sequence without noncoding region; * : genes located at the GT-rich strand; ?: stop codon not determined

Abbreviations: MG : *Meloidogyne graminicola*; PV: *Pratylenchus vulnus*; HG: *Heterodera glycine*; RS: *Radopholus similis*; BX: *Bursaphelenchus xylophilus*; BM: *B. mucronatus*; XA: *Xiphinema americanum*; EmtG: Entire mitochondrial genome; AA: Amino acid, bp: base pair.

Table S3. Measurements of 12 females and second-stage juveniles (J2) of *Meloidogyne graminicola*. [all measurements in μm and in the form: mean ± SD (range)].

Character	Female	J2
Body length	562.1 ± 52.5(464.7 - 693.2)	432.9 ± 25(369.7 - 461.6)
Stylet length	11.9 ± 0.4(11.4 - 12.3)	11.5 ± 0.7(10.2 - 12.3)
DGO	2.9 ± 0.4(2.5 - 3.5)	3 ± 0.2(2.5 - 3.2)
Anterior end to S-E pore	25.4 ± 2.6(20.9 - 28.2)	71.7 ± 4.9(60.4 - 79.1)
Metecorpus length	37.2 ± 2.4(34.1 - 41.9)	-
Metacorpus diameter	32.4 ± 2.4(27.8 - 36.3)	-
Neck length	146.8 ± 35.1(101.2 - 229.1)	-
Tail length	-	59.2 ± 7.5(47.6 - 68.9)
Tail terminus length	-	18 ± 1.7(15.6 - 21)
Anus to primordium	-	128.9 ± 12.1(115.8 - 150)
a	1.7 ± 0.4(1.3 - 2.4)	30.5 ± 2.7(25.1 - 35.1)
b	-	4.7 ± 0.3(4.3 - 5.3)
b'	-	2.3 ± 0.2(2 - 2.6)
c	-	7.3 ± 0.6(6.3 - 8.2)
c'	-	6 ± 0.7(5.1 - 7.5)
(S-E pore/L)*100	-	16.6 ± 0.7(15.3 - 17.6)

S-E pore : Secretory and excretory pore; DGO: Distance of dorsal pharyngeal gland orifice to style knobs.

Table S4. Information about PCR primers used in this study.

Primers	Sequence (5′–3′)	Source	Expected size of PCR product
COIF	GATTTTTTGGKCATCCWGARG	He *et al.*, 2005	400 bp
COIR	CWACATAATAAGTATCATG		
C2F3	GGTCAATGTTCAGAAATTTGTGG	Powers and Harris, 1993	1600 bp
MMT2	ATAAACCAGTATTTCAAACT	Harris *et al.*, 1990	
COILF1	GGTTTTATTGGTTGTTTAGTTTGAG	This study	6 kb
16SLR1	CTATGCTACCTTTGATCAATCACGCT		
COX2F2	AGAGCTATTACTTTAACTCGAGTCCAT	This study	15 kb
COIR2	TATAGCCGAGTTATCTGGTACGTAATT		

The IUPAC codes were used for K (G, T), W (T, A) and R (A, G).

Table S5. Species and GenBank accession numbers used in phylogenetic analyses in this study.

Species	GenBank accession number
Agamermis sp. BH-2006	NC008231
Ancylostoma caninum	NC012309
Ancylostoma duodenale	NC003415
Anisakis simplex	NC007934
Ascaridia galli	JX624728
Ascaris suum	NC001327
Brugia malayi	NC004298
Bursaphelenchus mucronatus	NC021120
Bursaphelenchus xylophilus	GQ332424
Bursaphelenchus xylophilus BxPt19SCD	JQ514068
Caenorhabditis briggsae	NC009885
Caenorhabditis elegans	NC001328
Chabertia ovina	NC013831
Cooperia oncophora	NC004806
Cucullanus robustus	GQ332426
Dirofilaria immitis	NC005305
Enterobius vermicularis	NC011300
Haemonchus contortus	NC010383
Heliconema longissimum	GQ332423
Heterodera glycines	HM640930
Heterorhabditis bacteriophora	NC008534
Hexamermis agrotis	NC008828
Loa loa	NC016199
Mecistocirrus digitatus	NC013848
Meloidogyne graminicola	KJ139963
Metastrongylus pudendotectus	NC013813
Necator americanus	NC003416
Oesophagostomum dentatum	NC013817
Onchocerca volvulus	NC001861
Pratylenchus vulnus	GQ332425
Pristionchus pacificus	NC015245
Radopholus similis	NC013253
Romanomermis culicivorax	NC008640
Romanomermis iyengari	NC008693
Romanomermis nielseni	NC008692
Setaria digitata	NC014282
Steinernema carpocapsae	NC005941
Strelkovimermis spiculatus	NC008047
Strongylus vulgaris	NC013818
Strongyloides stercoralis	NC005143
Syngamus trachea	NC013821
Teladorsagia circumcincta	NC013827
Thaumamermis cosgrovei	NC008046
Toxocara malaysiensis	NC010527
Trichinella spiralis	NC002681
Trichostrongylus axei	NC013824
Trichuris trichiura	NC017750
Trichuris suis	NC017747
Wellcomia siamensis	GQ332427
Wuchereria bancrofti	JQ316200
Xiphinema americanum	NC005928
Lithobius forficatus	NC002629
Limulus polyphemus	NC003057
